# Alterants

**Published:** 1838

**Authors:** John P. Harrison

**Affiliations:** Professor of Materia Medica in the Cincinnati College


### Art. II.—Alterants.

*An Essay on Alterants.* By John P. Harrison, M. D., Professor of Materia Medica in the Cincinnati College.

The term alterant, or alterative, is derived from *altero*—to change—and is employed to designate a mode of operation, rather than a particular medicinal article. Thus we familiarly talk of the alterant action and results of the various preparations of mercury; but that great agent is still arranged in our works on materia medica, under a different head. There seems to attach to the mode by which the alterant agency of mercury is effected, much of that mysterious influence upon which physicians, in former periods of medical history, were so fond of dwelling. The allusion has reference to the opinion universally entertained, in the days of an exclusive humoral pathology, that the alterant power of a medicine was resident in its capacity to accomplish some change in the circulating mass, by which mutation of the morbid state of the blood, the curative results were obtained. Whilst we retain the name of alterant, we attach not the slightest importance to the philosophy of the mode of operation, which was once so fashionable in the profession. On this point we desire to avoid all indefinite, intangible abstractions;—plausibilities are not to guide us in any matters pertaining to a physical science. Medicines operate as relative agents, and to appreciate their modes of operation, we must contemplate the varying conditions of the animal economy in which they are exhibited. To aver that alterants change the chemical constitution of the blood, or that they modify in some way its vitality, or that they correct morbid materials, by direct contact, in the current of the circulation, or that they reach distant parts by being absorbed, and by the channels of the circulation are enabled to exert their direct local action on diseased organs, may be a very facile method of ridding ourselves of all necessity for additional inquiry into the steps of the process by which the ultimate remedial influence is made to bear upon morbid conditions of the functions and tissues. By as rapid a march of conclusion we may refer the whole thing to sympathy, and thus without any more pains-taking in our investigation of the matter, we satisfy our minds that we have arrived at truth, when we have only imposed on ourselves a crude and hasty explanation for a minute and faithful enunciation of the phenomena.

In prosecuting this discussion, the first point of inquiry, to the elucidation of which we shall direct our attention, is, not *whether* alterants act by a mechanical force, or a chemical re-agency, exerted upon the blood, nor *whether* they operate merely by sympathy, but *how* they influence the vital powers—*what* therapeutic results flow from their judicious administration, and *in what* morbid states of the organism they are applicable. We desire to come up to the scrutiny of this interesting practical point divested of all partisanship—if ever such a feeling occupied our minds—and to give due weight to every accurately observed and faithfully recorded fact, which bears upon the question. And whilst we are determined to treat it as a grave practical question, we wish to allow no prejudices of the past, nor prepossessions of the day, to annul the decisions of a sound and authentic experience. It is obvious, upon the slightest reflection, that there exists no kind of mechanical relation between the remedies which we employ as alterants, and those states of the body in disease for the cure of which we prescribe them. And were we to concur, to the fullest extent, with the views entertained by former pathologists, that diseases were induced by alterations in the shape and size of the globules of the blood, still we could not perceive upon what ground an alterant was given, unless it were prescribed to act by a direct mechanical force upon the blood. But such a solution would ill comport with the spirit of an inductive logic. And as it respects the chemical theory of peccant matters, and of their neutralization in the blood by alterants, the same remark would obtain.

I. *How do alterants influence the vital powers?* First, in a way that is not accompanied by sensible evacuation; secondly, in a way which does not disturb the regular procession of the functions and actions of life; and thirdly, in a way which is subversive of anormal, or deranged action, by a process of either suspension, alteration, or substitution of action.

The principal idea attached by the profession generally to the term alterant is that of a curative power exerted by any remedial article, independent of any displays of its sensible action, by evacuation, or otherwise, on the system. An alterant is given with this indication in view—to so modify, change, or interrupt the morbid processes going on in the structures of the body as to bring about a solution of the disease, without disturbing the animal economy by any urgent solicitation of influence. It cannot, however, be denied that there may arise in the course of the administration of alterants symptoms of disturbance in one or more of the functions of life. Thus when mercury is given as an alterant, we may have several modes of its activity displayed, independent of its alterant agency. The simple excess of its peculiar alterant power may create extraordinary excitement in the glandular system. The salivant action of mercury is separable from its simple alterant action. But the salivation resulting from the remedy is neither a proof of the injurious activity of the mineral, nor of its curative power. The kind of salivation induced is more to be depended on than the degree of irritation created in the glands of the mouth. Still, as the general capabilities of mercury in the arrest of disease is not measured by its salivant influence, we may adhere to the definition, and insist on the precision of our view of the modus operandi of an alterant, when we contend that it is an agent of the materia medica, which acts curatively, independent of any sensible disturbance in the functions of the body. Primordially, the action of an alterant is upon the vital properties of the system, in virtue of the relation which exists between the state of those vital properties, and the article administered.

It is only by a careful generalization from a great variety of well conducted trials, and the results of those trials, in diversified morbid states of the living human body, that we derive a just experience of the virtues of any medicinal substance. From deductions, drawn from a wide field of experience in the profession, we are warranted in referring the peculiar power of alterant medicines to a special medication which they create. We do not believe in specifics of any sort,—not that certain remedies bear no direct or special relation to certain organs, or certain conditions of those organs, but because there can be no such thing recognised in scientific medicine as a disease, abstractly considered. All diseases consist in certain conditions of the organism. To suggest that a remedy is a specific for a disease is to do violence to the first principles of a just pathology. Alterants act in a special manner on certain organs, and on certain states of those organs. Their action is one that is independent of sudden exaltation, or immediate perceptible depression of the functions of the organs upon which they exert their activity. It is an activity, not revealed by a more rapid circulation quickly created in the system, nor by a direct augmentation of vital force in any part of the body, nor is it an activity that shows itself by a subversion or disturbance of the accustomed actions of life.

Incidental, or consequential, to the power possessed by alterants in so modifying the morbid conditions of the organs as to subvert anormal processes going on in them, there are various evidences of a more energetic and harmonious movement established in the vital endowment of the organic structures. These evidences of a renewal of the healthy state of the parts originally diseased, or of the sympathizing portions of the frame, should be placed among the collateral and resulting phenomena, which accompany, or spring from, the primary alterant action.

The therapeutical action of alterants is evinced, 1st. by suspension of the morbid phenomena; 2d. by the superinduction of a new action; and, 3d. by substitution of action. Let us illustrate these points. All the morbid phenomena witnessed in the human body may be arranged under the respective heads of constitutional and local, as respects the extent of the action originating them; and of functional and structural, as regards the kind of action producing the phenomena; and acute and chronic, in reference to the period of their duration.

A constitutional affection often gives rise to local diseases, and local disordered action frequently produces constitutional derangement.

Functional disorder may exist without lesion of structure; but often structural changes are induced by disordered function. Mr. Abernethy seems to limit the use of alterants to their utility in rectifying vicious states of the chylopoietic viscera, but Sir Wilson Philip has extended their interference to cases of functional and structural diseases of various important tissues and organs.

The action of alterants is often seen in suspending morbid states, and thus permitting the powers of health to resume their wonted condition. This we have often exemplified, after the exhibition of minute doses of calomel in deranged conditions of the alimentary tube. The same mode of operation is evinced by minute doses of corrosive sublimate in diseases of the skin, and by other mercurial preparations, when given in small quantities, in affections of the various organs. The gentle, quiet, and undisturbed curative interposition of alterants, in many functional and structural affections, to be hereafter noticed, depends upon the ability of this class of medicines to suspend morbid processes.

The therapeutical advantages to be secured by alterants is further seen by their power in arresting diseased actions in consequence of the modification which they impress on constitutional and local, functional and structural, acute and chronic affections.

In the constitutional treatment of local disease, we often proceed upon this recognized capability in our alterant remedies. Thus in iritis, in hepatitis, in red hepatization of the lungs, we administer mercury in order to produce an alteration of the action giving rise to the deposition of coaguable lymph. Iodine is given in strumous affections, with a similar indication in view. Arsenic is administered in some diseases, with a like intention.

Substitution of action is another mode in which alterants act in the eradication of disease. When we produce a mercurial impression to cure fever, we substitute the action of the remedy for that of the disease. When we create a strong impression upon the functional activity of one organ, such as the salivary glands by mercury, or on the kidneys by hydriodate of potash, we bring into requisition a subsidiary action to aid the other two modes of agency exerted by alterants. There is, however, a manifest limitation to this last mode of operation. If it precedes, or surpasses, the other methods of action instituted by alterants, our final design may be frustrated. The action of substitution, the metaptosis, or metastasis, of the older writers, is often exhibited in disease. And the therapeutist will avail himself of this law of morbid action to substitute an artificial, definite, and controllable constitutional action, for one that is anormal, unlimited, and not corrigible by any power of the system.

These three modes in which alterants exert their benign control over disease are often seen conjoined in the effects brought about by our greatest alterant, mercury. But these several influences exerted by mercury, according to its different preparations, and extent of application, should not be confounded. There is a gradual alterant influence exercised by very minute doses of milder preparations of mercury. And there is a more energetic power displayed by the proto-chloride, and the oxides of the metal, when pushed to a full issue, which we can never bring about by the stronger preparation.

When we wish to secure the full effects of the two first modes of agency of alterants, that of suspending morbid action, and that of inducing a new action, we should guard against a powerful local impression. Remote changes do not readily ensue upon the exhibition of alterants, when the organ with which our remedy is brought in contact feels its direct impression. Nor should we persist in the use of an alterant when any sudden irritation, or disturded movement, succeeds its introduction into the stomach. If we give mercury, iodine, or arsenic, as alterants, and find that our medicine nauseates, or purges our patient, we must desist from the employment of it. And if after a few days, constitutional irritation arise, or the mouth becomes very sore, or pain arises in the abdomen, we must cease the use of the article. Alterants act often as local remedies. We frequently exhibit small doses of calomel in deranged conditions of the mucous tissue of the intestinal canal, to alter morbid action set up in that surface. In dyspeptic affections, ipecacuanha is often employed with a similar design. Iodine is used as a local remedy, to disperse grandular enlargements.

Alterants are either anti-inflammatory and anti-febrile, or tonic and exciting. Mercury, ipecacuanha, and tartar emetic are often made available to the solution of febrile and inflammatory diseases.

Iodine is in possession of powers of a tonic and exciting kind, in connection with a special alterant agency.

Arsenic is not a tonic in any just sense of the term, but is an exciting alterant.

II. *What are the therapeutical results which are seen to arise upon a judicious administration of alterants?*

Diseases are, we have said, either constitutional or local; that is, they affect either the whole organism, or they affect only portions of it. In constitutional diseases alterants have achieved their most conspicuous curative triumphs. The dominion exercised by alterants over morbid states of the general system has been attributed to the direct modifications which they are capable of imparting to the molecular movements of the blood in the terminal branches of the vascular apparatus. By others, their controlling influence has been ascribed to their primary operation upon the blood; and according to the views of some eminent physicians, their primary impression is made upon the nervous system, and by sympathy this impression is radiated to all parts of the organism. We candidly confess, that we know but little about the manner in which they induce, or bring about, those curative sequences which we every day witness after their exhibition. That alterants exert a strong modifying influence upon the capillary system, we are ready to admit. The modifications of secretory function which follow their use, are evidence of their salutary office in morbid conditions of the capillary system of the body. The increased temperature of the surface, with an expanded pulse, and a better hœmatosis, are proofs of their power on the blood, and the moving forces of the circulation. The abatement of constitutional irritability, and the mitigation, or removal of deranged sensations in different parts, such as the head, chest, abdomen and limbs, by their employment, demonstrate that alterants act on the nervous, as well as general vascular and capillary systems. The direct interference of mercurial action in putting a termination to fever and inflammation, shows us, conclusively, that we must not take any exclusive, restricted view of the modus operandi of the great alterant of the materia medica. Why should we so keenly contest the comparatively unimportant question of the introduction of alterants into the current of the circulation. Admit for a moment that mercury, iodine, tartar emetic and arsenic do pass unaltered into the blood. The question is still unresolved, why they exercise such a diversity of therapeutical actions. These ultimate facts are admitted on all hands—that no two efficient substances in the materia medica act alike—that one article displays a peculiar aptitude to fulfil one indication—and whether we admit the truth of the position or not, that they do veritably penetrate the barriers set up by nature for the exclusion of foreign matters into the vital fluid, we all must come to the same conclusions, derived from an experience of the benefits flowing from the use, and of the injuries apt to spring from the abuse of these our most energetic medicines.

When employed within the boundaries of a gradual and gentle medicating influence, alterants cannot be said to interfere with the ordinary functions of the economy. Iodine, given in small doses, quickens the appetite, and tends to the production of a more successful nutritive secretion. When pushed to the extent of superinducing *iodism*, as it has been called, when symptoms of gastric and constitutional irritation arise on its administration, rapid emaciation will ensue. Mercury and tartar emetic, when given in minute quantities, do not disturb any of the functions; but when a decided constitutional agency is exerted by them, we have, with a more redundant emission of secretory products, a dimunition in the nutritive depositions of the system. Arsenic evidently lessens the tone of the stomach, but acts by imparting excitement to the cutaneous vessels; but this excitement is essentially different from a mere exaltation of the physiological condition of the dermoid tissue. It is an excitement subversive of certain chronic ulcerative and eruptive affections of the skin, which are not amenable to a simple increase of physiological excitement.

III. *Therapeutical applications of Alterants.*—In constitutional maladies, or in local disorders, connected with constitutional derangement, alterants are most frequently exhibited. Constitutional diseases may be either acute or chronic, febrile or inflammatory, cachectic or scrofulous.

Constitutional disturbance may produce local disease, or be induced by it. In the majority of chronic complaints, there is the precedent existence of disordered function in the stomach, duodenum and liver. Sometimes disordered function commences in the skin or kidneys; but there are few cases of a decided, predominant, primary functional irregularity of either the skin or kidneys. The latter organs are so isolated from external causes of disease, that they seem almost incapable of originating morbid action. The renal function is subsidiary, and in part vicarious, of the office of the skin, and is in close dependency to the chylopoietic viscera. Deranged digestive function is generally the source of irregularities in the functions of the kidneys.

The brain, in civilized society, is unduly excited, and liable to disordered function, which may precede changes of structure in the cerebral mass. But the brain rarely suffers abiding mischief unless the functions of the abdominal viscera become disturbed. In this connection we have not an exclusive reference to those kinds of disease which are detectible upon a slight examination of the functions of the sub diaphragmatic viscera. The brain, lungs, abdominal canal and subsidiary organs, and the kidneys, may each become functionally disordered, and finally diseased structurally, by some general diathesis, of either an acute or chronic character. Idiopathic fever we hold to have a substantive existence. Its series of phenomena have been well delineated by Dr. Southwood Smith, and to his able work we would refer for an explicit and satisfactory exposition of those lesions of innervation, circulation, secretion, and of nutrition, which occur in fever. Struma is a constitutional affection, not a disease of any one allotment of the tissues, such as the lymphatic system, but a morbid condition of the constitution, which is almost omnipresent; for it is liable to seize upon nearly every organ and texture of the entire body.

We cannot trace the origination of this malady to any one department of the tissues; nor can we localize it primarily in the digestive apparatus, as some distinguished writers have done. The venereal disease is another constitutional affection. Mercurial is another. Gout and rheumatism, especially when hereditary, are clearly constitutional maladies. In all these general, or constitutional diseases, we employ alterants. And in the treatment of those topical affections, which are grafted upon these different diatheses, we call into constant requisition alterant remedies. In acute, as well as chronic, inflammatory complaints, alterants are liberally employed. In arachnitis, peritonitis, pneumonitis, and various other inflammatory diseases, we avail ourselves, after free sanguinous depletion of the alterant agency of tartar emetic and calomel. The power which tartar emetic possesses in suspending febrile and inflammatory action is clearly dependent upon an alterant, and not upon a contra-stimulant agency. The tolerance of the stomach for the remedy, and its very limited sensible action in producing evacuation of any kind, show us conclusively that it exerts a special alterative influence upon the morbid processes set up.

Fordyce, in his work on fever, attributes the beneficent effects of minute doses of tartatized antimony in fever to its febrifuge operation, which he asserts is entirely separated from its evacuant properties. "Preparations of antimony and ipecacuanha, administered so as not to produce vomiting, bring on appearances similar to those of crisis in fever. When the stomach in fever will bear but a very small dose without producing sickness, (as one-fifth or one-sixth of a grain of tartar emetic, for instance,) the author has rarely found that any critical symptom has been produced." Such is the language of this distinguished author, who contemplated fever with an eye of penetrating discernment, and who has given us a most graphic and faithful delineation of its symptomatology; but whose treatment partook largely of the expectant method.

Dr. Chapman, in his Materia Medica and Therapeutics, entertains a similar opinion in reference to the powers of antimonial preparations in fever. He thus speaks, in relation to the administration of this remedy in fever:—"Medicines seem to do good in the cure of fever, by exciting their own peculiar or specific action. When they disorder the stomach by sickness, they depart from this, and if they do not act as poisons, always become nugatory, or more or less mischievous. Nausea, by whatever means induced, is not, in itself, a salutary effort, nor does it ever dispose fever to a crisis, or favorable solution."

We cannot go with Dr. Chapman to the extent of his views respecting the induction of nausea. We believe that when the febrile action is high, the production of nausea by tartar emetic, instead of proving nugatory or mischievous, exercises a favorable control over the irregular and disordered excitement of the system.

Marryatt, an eccentric physician, who published a small treatrise on therapeutics, the 21st edition of which was printed in 1790, thus testifies to the alterant action of antimony in fever. "Any fever may be soon extinguished by the use of the following powders:—take of tartarized antimony five grs., white sugar (or nitre) ʒi; let them be well rubbed in a glass mortar, and divided into six powders; one to be taken every three bours, notwithstanding the nausea the first may possibly occasion. If these are taken (which is commonly the case) without any manifest inconvenience, let there be seven grains in the next six powders; and in the next ten. Here I beg to retract what I said in some former edition of this work, viz. that till sickness and vomiting were excited, this noble medicine was not to be depended on. For I have since seen many instances wherein a paper has been given every three hours, (of which there has been ten grains in six powders) without the least sensible operation, either by sickness, stool, sweat, or urine, and though the patients had been unremittingly delirious for more than a week, with subsultus tandinum and all the appearances of hastening death, they have perfectly recovered without any medical aid, a clyster every day excepted. I have lately seen a great many cases similar to the above, and the tartarized antimony has invariably produced the same effect."

The combination of small doses of calomel and tartar emetic, with the nitrate of potash, in fever, and in the phlegmatiæ is of superior excellence in bringing about a reduction of the force of morbid action, and in arresting lesions of structure in the important organs. The excitement must, however, be brought down, when intensely exalted, by the lancet and other depletory means, before this combination can be made to secure the desired alterant result. The restoration of the secretions of the liver, skin, and kidneys, is one of the most signal manifestations which we witness, as the incidental and consecutive phenomenon, arising upon the use of the above combination. This triple union of articles, so decidedly alterant, does not owe its valuable febrifuge and anti-phlogistic properties to the evacuations which may follow its exhibition. We have already declared our conviction, that alterants are in possession of a three-fold mode of agency;—the power of restoring the secretions exerted by the combination referred to, is collateral to the power of suspending morbid processes, and of creating an altered action. The pulvis antimonialis and ipecacuanha possess alterant properties inferior to tartar emetic in febrile and inflammatory seizures. But either may be advantageously given in certain forms of reduced febrile and inflammatory excitement.

When there is an obstinate persistence of fever, or of acute inflammation, threatening the destruction of some vital portion of the system, by the supervention of a rapid disorganizing change in the structure of the parts affected, our final resort is to mercury. Speculative plausibilities may safely be indulged in as long as they do not cloud our discrimination of disease, and intercept the full application of decisive measures for the relief of the sick. But let the ultraist in medicine beware how he decides *a priori* that fever is a simple and easily governed form of morbid action, dependent upon a primary inflammation of some viscus, and that to arrest fever, all the physician has to do, is to ascertain the point of original disturbance, and subdue the disordered state of that suffering organ. Fever is a very complicated affection, and is by no means amenable to any exclusive mode of medication. But of all the remedies which chemical science has conferred upon the art of healing, there stands no single article so pre-eminently endowed with a diversified capability of curing disease as calomel. Assuredly no remedy, with which we are acquainted, exerts a thorough alterant agency equal to this great medicinal substance. When we declare that its powers are unique and unrivalled, we only embody the general testimony of the profession in its favor. No medicine is as frequently appealed to in the southern and western parts of our country, for the cure of fever, as this effective and common preparation of mercury. And although its friends have often proved its worst foes, from the misguided and indiscriminate manner, and abusive extent, in which they have exhibited it, still it survives the assaults of its enemies, and the perverted applications of its idolatrous admirers, as one of our surest, most decisive, and effective agents in the cure of severe febrile and inflammatory attacks.

Dr. Farre, in his interesting communication, addressed to Mr. Travers, contained in Cooper's & Traver's Surgical Essays, gives the following suggestions on the therapeutical action of mercury: "Whilst the principal tendency of the series of remedies, which we comprise under the received term, anti-phlogistic, from general blood-letting downwards, is to diminish the force of the heart and arteries; it is in a peculiar manner the operation of mercury on the whole capillary arterial system to change its action, but not indefinitely. I had been led, from repeated observation of the adhesive inflammation of various textures being cured by the mercurial action, to receive it as one of the *general laws* of its operation to change the arterial action on which the effusion of coagulable lymph depends, and consequently to arrest all the subsequent changes which flow from this process."

This explanation of the modus agendi of mercury, we think, is not to be taken without some qualification and abatement. For mercury will, when unduly urged, often create an inflammatory irritation, in which coagulable lymph may be poured out. That mercurialism is productive of a species of constitutional disturbance, involving both the nervous and vascular systems, we see exemplified every day. The alterant powers of the remedy may be exercised without this frequent accompanying disturbance of the constitutional energies.

Mercury may be employed in acute as well as chronic maladies, in such a way as to accomplish all its curative ends with little or no sensible evidences of its alterative agency. This point, we confess, is not entirely under our mastery; for often, very unexpectedly to us, the severe salivant and perturbating effects of the remedy are revealed, after the corrective operation of it on the disease has been attained. In the treatment of ordinary fevers, of inflammations of the different organs, and of syphilis, there is no necessity that mercury should be pushed to an urgent and intense impression on the system. In proportion to the aggravation of the deranged and perverted actions set up in the system, and its component tissues, must be the boldness and decision of the practitioner in the use of mercury. But a sore mouth does, by no means, constantly attest the beneficent power of the remedy upon the constitution; for a misplaced and mischievous inflammation may occur in the mucous coat of the cheeks and fauces, in cases of disease in which a mercurial action has been attempted. The practical advantages of mercury in acute affections flow from its excellence as a promoter of the secretions of the whole secrenent organs of the abdomen, as well as from its superior alterant operation in arresting disordered function and structural changes. In the three great splanchnic cavities, the head, thorax and abdomen, inflammation is often lodged. These containing cavities, are filled with viscera, which are liable to various degrees and kinds of derangement; and the adjuncts, or investing and interposed tissues of these important viscera, are likewise often the seat of severe morbid processes.

Lesions of secretion and lesions of nutrition often destroy life by encroaching on the integrity and vitality of these organs. Thus we are taught by pathological anatomy, that effusions of pus, serum, and coagulable lymph are the frequent terminations of inflammations in the brain, thoraric viscera, and abdominal organs.

And thickening, induration, softening, and ulceration, are often seen as the modes in which the severe morbid actions of these parts come to a disastrous conclusion. The alterant powers of mercury are never to be overlooked in our treatment of such cases of disease. This medicine is often competent to reach and subdue such affections; and although not always successful in the employment of it, yet there remains a large residue of certainty and effectiveness in our experience, after the most ample deductions for incorrigible cases, as to justify and embolden us in the further use of the remedy.

We wish not to be misunderstood on such a grave practical point as that under consideration, and we therefore distinctly state, that we place the use of tartar emetic and mercury, as alterants, as subsidiary and co-operative to the general and topical evacuations of blood, and other methods of reduction, in severe febrile and inflammatory complaints. But after a reduction of the moving forces of the circulation by our evacuant remedies, we advert, with strong hopes of a happy issue, to the exhibition of these excellent medicines. Fever, inflammation, and syphilis have been often cured without any alterant. But this affords no sound reason for their judicious employment, as we are well convinced, that on the ground of an enlightened and humane expediency, it would not be proper to pretermit their administration.

In chronic diseases, alterants are chiefly employed. The extensive sway exercised by the chylopoietic viscera, in inducing disordered states of the thoracic viscera, has long been recognized, and made the basis of therapeutical interference. The encephalon is likewise very often brought into a morbid condition, by the disturbing influence exerted upon it by abdominal irritation. The general nervous system seems peculiarly alive to the abnormal actions going on in the organs of nutritive life. The skin and kidneys are, as we have already observed, often thrown into morbid conditions by the disordered functions, or structural changes, of the chylopoietic viscera.

We will bestow a brief consideration on the related diseases of the three great containing cavities, and deduce from our examination of the connections and modes of communication, of the morbid actions of the encephalon, thoracic viscera, and abdominal organs, some inferences respecting the exhibition of alterants in such related affections.

Diseases, generally, may be related, 1st, by predisposition; 2d, by etiology; 3d, by the seats they occupy; 4th, by extension; 5th, by remote changes; and, 6th, by conversion, or substitution.

1. Predisposition may be either hereditary, acquired, ætal, or sexual. That many chronic maladies, affecting different parts of the system, have their substratum in the hereditary proclivity, or tendency of the constitution, is universally admitted. Struma is a disease of transmission from parents to children; and such is the strong bias to this peculiar form of morbid action in some families, that when any other kind of disease is established in the system, that new form of morbid action is soon modified by the previous existing tendencies of the organism to scrophulous derangement. All the organs and tissues are brought under the dominion of this strong predisposition; and no matter what species of anormal movement arises in the system, the subsequent phenomena partake much of the nature of this strumous diathesis. Tubercular deposits often occur, in such instances, in various parts of the body.

The same relation exists between affections of the brain, where a hereditary predisposition reigns in different members of the same family. Chorea, epilepsy, and madness are thus related. One member of a family will have chorea, one epilepsy, and another madness, or there will be an alternation of these affections in the same individual. Dr. Parry, of Bath, has given some very apposite instances of this kind in his work on pathology, and in his posthumous writings, published by his son, others are communicated.

Acquired predisposition is as familiar to the eye of the experienced and observant physician as a hereditary tendency to disease. The tubercular cachexia may be hereditary, or it may originate from modes of living. A predisposition to phthisis pulmonalis is often generated by a too rigid and abstemious regimen. During the prevalence of the epidemic cholera, we knew persons who brought on a fatal tendency to a tubercular formation in the lungs by their pertinacious adherence to a course of living too reducent and attenuating.

Paludal exhalations induce in man a state of predisposition to certain forms of febrile disorder. This state of predisposition is called by some authors the latent period; by others, the stage of incubation, and may be regarded, pathologically, as a stage of formation, in which the system lies under the power of a remote cause, without sensible disturbance of its powers; or if not entirely dormant, the morbific agent has not acted so energetically as to evolve urgent and well marked febrile phenomena. When brought under a predisposition to bilious fever, there exists an assimilating character about the system, which blends hepatic derangement with every morbid action, however induced, that may be set up. This is exemplified in cases of pleural inflammation in persons living in a miasmatous district of country—the pleurisy is invariably complicated with symptoms of bilious fever. So familiar is this relation of diseased actions, that we designate the complication by the very popular title "bilious pleurisy."

Age and sex both produce certain predispositions, which we cannot notice with particularity.

2. The causes of disease induce intimate relations between morbid affections. The cause of epidemic maladies is some poison existing in the atmosphere. This agent acts on the vital endowment of the system, not simply as an excitant, or as a depressant, but a modifier of the living energies, perverting their normal operation. The related diseases springing from the impression of marsh miasmata, are frequently seen among us. Ague, mild remittent, inflammatory, and congestive bilious fever, with dysentery and hepatitis, are related to each other by paternity.

3. Diseases are related to each other by the seats occupied by them. An inflammation in one part of a mucous tissue, in virtue of the connection existing between the entire mucous surfaces, is very apt to spread to other portions of the same structure. And in rheumatism there exists a tendency in the disease to shift from one part of the fibrous system to another; and in consequence of this law of pathological action, the heart often becomes the seat of the transferred inflammation.

4. Diseases are related to each other by a pathological law of extension. The direct nervous communication existing between organs, their vascular relation, their functional union, and their contiguity to each other, are elements which constitute the law of extension, or epigenesis, in diseases.

5. Diseases are related to each other by conversion. Substitution of morbid action may arise in several ways. An irritation existing in the chylopoietic viscera may induce irritation in the lungs; this irritation may then cease, and the secondary, or resulting malady, pursue its fatal march. Or a morbid action may be suddenly suppressed in one part, and a diseased state of another part rapidly spring up. This we often see in the sudden retrocession of eruptive maladies.

6. Diseases are related to each other by remote changes. Thus a morbid state, having affected one part at one time, is disposed, in some persons, to seize on other parts, at another time. Hemorrhoids, hemoptisis, diarrhœa, and cutaneous eruptions, succeed each other in some persons. Dropsy, followed after a short time by rheumatism, and that succeeded by gout, have been seen. "In several instances," says Dr. Parry, "I have seen fits of epilepsy wholly superseded by those of gout. In one patient, the succession, at somewhat remote periods, was gout, mania, and, at last, fatal epilepsy."

In conducting the treatment of chronic affections by the administration of alterants, it behoves us to keep in view these relations and connections of morbid action. The empirical employment of any class of medicines is most earnestly to be avoided by the enlightened physician. No arbitrary juxta-position between the name of a malady and its appropriate means of cure should exist in his mind. In our endeavors to arrive at just conclusions in reference to the true indications of cure in disease, we should take into consideration the above-mentioned points of pathological inquiry.

To particularize,—let us investigate the several modes by which disordered function in the digestive apparatus leads to derangement of sensation, function, and action in other parts of the system, and then individualize the forms of disease in which alterants, by correcting this state of disordered function in the chylopoietic organs accomplish the cure.

The morbid states of the abdominal viscera may produce disturbance of sensation and function, and even lesion of organization, in the thoracic contents, in the following modes:—1. Through nervous communication established between the pneumo gastric and the central ganglia of the sympathetic nerve; 2. through vascular connection; 3. through similarity of texture; 4. through functional union; 5. by contiguity of organs. In the first mode, we have cough excited by the presence of worms in the alimentary canal. An instructive case is given by Dr. Graves, of Dublin, in his excellent clinical lectures, which reflects directly on this point. A young lady in Dublin was attacked with fits of coughing, of extraordinary intensity; the cough "was dry, extremely loud, hollow and repeated, every five or six seconds, night and day, when she was asleep as well as when she was awake. Its violence was such that it threatened, to use a vulgar, but expressive phrase, to tear her chest in pieces." There was no fever in the case, and "she did not fall away proportionally in flesh, though bled, leeched, blistered, and got the tartar emetic mixture, but without experiencing the least relief." Opium, conium, hyoscyamus, and Prussic acid, were had recourse to, without the slightest benefit. Her physicians gave up the case in despair, and she was cured, *all at once, by an old woman.*" This veteran practitioner, a servant in the family, suggested the exhibition of a large dose of spirits of turpentine, with castor oil, for the purpose of relieving a sudden attack of colic; two or three hours afterwards, the young lady passed a large mass of tape worm; and from that moment every symptom of pulmonary irritation disappeared." Irritable dyspepsia, in which there exists a morbid sensibility of the stomach and intestines, is apt to induce bronchial irritation and pain, with fits of palpitation of the heart.

Vascular connection unites in intimate relation the morbid states of the abdominal and thoracic viscera. A reflux of blood occurs in the venous system, in cases of aneurismatic dilatation of the heart, which is productive of engorgement of the liver, and of gastro-intestinal irritation, and hyperœmia of the mucous tissue of the digestive tube.

The mucous tissue diffused trough the bronchi and alimentary canal binds these parts together, in morbid as well as in physiological conditions of the viscera of the thorax and abdomen.—This is exhibited in cases of croup, in which there exists inflammation of the mucous tissue of the lungs, with great insensibility of the internal coat of the stomach. Asthma often arises from irritation of the internal surface of the stomach and bowels. Protracted inflammatory dyspepsia brings on chronic bronchitis.

The liver and lungs are regarded by some eminent physiologists as co-operative organs in the production of certain changes in the blood; but we think that this speculation is hardly sustained by well generalized facts.

The diaphragm is interposed between the two great cavities; and the liver, when inflamed, produces irritation in the lungs, by the relation of contiguity. Suppuration of the liver is often terminated by a discharge of pus through the diaphragm and lungs.

Disordered function of the chylopoietic viscera is met with under several familiar aspects. Dyspepsia is the most common. This may be atonic, irritable, inflammatory, or follicular, and may be radicated primarily either in the stomach, duodenum, or colon. These several forms of dyspepsia engender a morbid irritability of the innervation, with more or less disturbance of the vascular system. This constitutional irritability, with irregular distribution of the supply of blood to the different parts of the body, brings on a train of morbid phenomena in the head, lungs, skin and kidneys. Deranged sensations in the brain, such as hallucinations of mind, illusive impressions, and sleeplessness, often are precursors to melancholia, terminating in madness or suicide. The lungs are thrown into asthmatic efforts, and the heart is irritated into palpitations. The kidneys secrete new products, and new combinations and proportions of the original elements of the urine.

Urinary calculi and saccharine urine are often the products of a disordered state of the functions of the assimilating organs.

Hepatic derangement follows, or co-exists with the interrupted or perverted functions of the stomach and bowels. This biliary disorder contributes still further to the deranged condition of the constitution and of the brain and lungs. Grafted on peculiar states of predisposition in the system, these conditions of the gastric, intestinal and hepatic functions bring on hydrocephalus or phthisis pulmonalis. Where ætal and scrophulous predispositions are conjoined with this engrafted, or superadded disturbance of the chylopoietic viscera, there is frequently engendered incurable lesions of secretion, or structure, in the encephalon, or thorax. Dr. Cheyne regards hepatic derangement as an essential element in the origination of dropsy of the brain. We think he has carried his view on that point beyond the warrantable bounds of a sound induction. Diseases of the skin are often traceable to disordered function in the organs of nutritive life.

Marasmus, connected with enlargement of the mesenteric glands, is not attributable to any mechanical impediment to the free course of the chyle through the lacteals, for experiments performed by Soemmering and others, have proved that no such obstruction exists as is generally supposed. The cellular texture around the chyliferous tubes is the seat of the irritation and inflammation, which destroy life, in fatal terminations of the disease.

In the disordered function of the bowels and liver, there sometimes supervenes a structural change of the mucous surface of the digestive tube, or of the organization of the liver. But these organic changes are quite infrequent, compared to the number of cases of merely disordered function.

There are four great organs in the system, which are constantly giving egress to the peculiar fluids of their secretory elimination—the skin, the liver, the mucous coat of the stomach and bowels, and the kidneys. These emunctorial outlets reciprocally affect each other. If either the skin, liver, intestinal canal, or kidneys, fail in their functional activity, and fall into a disordered condition, the others soon realize a derangement in their functions. In administering alterants we should keep this principle of pathology in view.

There are rational and physical signs to warn us of the states of disordered function in the intestinal canal. The tongue is not to be too exclusively relied upon in our diagnosis; for it frequently is coated when there is no special disturbance of the digestive passages. The state of the prolabia, a dry and fissured state of lips, with aphthous sores on the tongue, we often have in irritated states of the bowels. The deranged sensations, such as pain, sense of distension, and oppressive weight, about the præcordia, with the deranged functions, indicated by confined or very loose bowels, and unnatural alvine excretions, denote a morbid condition of the chylopoietic viscera. The pain and fulness so frequently present in the right side, are not to be too confidently relied upon as denotive of hepatic disturbance. Fœcal accumulation in the colon, and ulceration in lower part of the ileum, have been treated for liver disease, to the great injury of patients.

IV. *Rules for the administration of Alterants.*—There are general rules of therapeutics in the application of this class of medicines, which should receive a distinct and prominent notice at our hands. The first rule to be observed is, a due preparation of the system, for the reception, and appropriate agency, of the alterant we are about to administer. In acute maladies, this rule is of especial authority. In high inflammatory action, blood-letting should precede the use of our alterants. Where the constitutional powers are reduced, we should not institute the alterative plan of treatment till the constitution has been invigorated by a course of preliminary tonic treatment. This provisional measure is of paramount weight in the employment of mercury in cachectic, or broken down constitutions. We may speculatively expatiate on the stimulant action of mercury; but in the practical applications of this remedy we are taught, by experience, not to expect from it any benefit in cases of shattered and enervated vital powers. Where it is deemed of absolute importance to exhibit mercury to enfeebled patients, we should gradually employ it in combination, or cotemporaneously, with tonics. Mere debility, however, is not to deter us from the use of alterants of the anti-inflammatory or antiphlogictic character; for that debility may be but a symptom of some deep-seated inflammation.

There is then a preparation of the system which should be secured in reference not only to the excessive degree of contractile power displayed by the heart, with the corresponding development of animal heat, but likewise as regards the prostration of that power, with its corresponding reduction of animal temperature. On these most interesting points of practice we cannot dwell; but they are of such magnitude, and of varied applicability, in the treatment of acute and chronic disease, that they are deserving of expanded discussion. Too often have we witnessed melancholy results spring from the neglect of the above truths. Patients in fever, prior to due depletion, are often mercurialized by the injudicious administration of large quantities of calomel; and too frequently their lives pay the forfeit of this premature attempt to bring on the specific action of mercury.

Where the general powers of the system are prostrated by the suffocated excitement of fever, the phenomena present indicating what is generally called congestion, the mercurialization of the patient is often our best resource. This step is not always decisively useful; but it holds out, after counter irritation and moderate depletion, by cups over the chest, abdomen and head, our most effective means of preventing lesions in the great splanchnic cavities.

In chronic affections, there may co-exist either an absolute or relative plethora. Absolute plethora, the plethora *ad molem* of the ancients, is known by vascular fulness, strong venticular action, and other evidences of high health.

In relative plethora there exist partial determinations of blood to particular organs, with a constant tendency to congestive irritation, and suppressed, or disordered function, of the secretories. In both absolute and relative plethora, we must reduce action before we administer alterants. But by judicious combination of remedies, within certain limits, we may control the plethora, whilst we are conducting an alterative plan of treatment.

The second rule which should govern us in the employment of alterants is not to attempt the attainment of a too sudden result. Time is an essential element in the beneficent influences to be exercised by alterants. This remark has special relevancy to chronic diseases. To subvert long established disordered function, or to displace structural alterations, by this class of remedies, we must adapt the quantity given to the excitability of the body, and of the deranged organs, and protract the use till through a gradual, slow process, the morbid action is overcome by gentle solicitation rather than by severe aggressive medication.

Sir Wilson Philip informs us, in his treatise entitled "The Influence of Minute Doses of Mercury," that he has for thirty years employed minute and frequently repeated doses of mercury, and that he was led to this practice, "by observing that, in lessening the dose and increasing its frequency, in proportion as we lessen the immediate, we increase the alterative effects."

From our great familiarity with the more energetic displays of action which mercury and tartar emetic exert upon the system, we are very apt to underrate the more subdued and tranquil modes in which they are capable of accomplishing diversified curative ends.

Calomel and the blue pill are generally resorted to for the purpose of producing direct purgative results. Mr. Abernethy erred, we conceive, on this point, for although these valuable medicines are often called for to excite in a rapid manner the biliary function, and induce a more accelerated transit of fœcal matters through the alimentary canal, yet a too exclusive regard to these immediate obvious effects is calculated to mislead our minds from a due estimate of the excellence which attaches to these articles, when given in minute and frequently repeated doses.

The commonly employed preparations of mercury may act remedially, 1st, by their local alterant impression upon the digestive tube, and adjoining organs; 2d, by the institution of an increased secretory action on the mucous tissue of the bowels, and on the heapatic apparatus, create a revulsive movement, remedial of the morbid condition of other organs; 3d, a constitutional agency, partaking of a revolutionary character, evidenced by glandular activity, excited circulation, and nervous irritability; 4th, by an insensible, tranquil, alterative agency, which though subversive of diseased states of the organism, does not interfere with the regular operations of life. In chronic disease, this last result we should exclusively aim at. Mercury may act mischievously in the following modes: 1st, by proving an irritant of a violent kind to the stomach;—we meet in practice with persons who are thrown into excessive agony by a dose of calomel, or blue pill; 2d, by acting as a salivant;—there are persons who have been badly salivated, and who suffer from a renewal of the ptyalism by one grain of calomel, or a few grains of blue pill. But in the administration of mercury to persons who have never been salivated, and who do not labor under any idiosyncracy, we find occasionally anomalous symptoms springing from its employment. Erethismus and eczema mercuriale, with dry gangrene about the mouth, may come on singly, or in combination. We do not believe that sound observation warrants the opinion held by many in the profession, that mercury gets into the blood, and thence is conveyed to the bones. But that there is such a real, substantive malady as mercurial disease, we have had many opportunities of verifying. Now, the bold and reckless manner in which the remedy is employed, even in chronic affections, is eminently calculated to bring on a most afflicting and incorrigible constitutional disease, which often defies the skill of the most experienced and enlightened physician to cure.

The next rule of importance in conducting the treatment of diseases by alterants, is to combine them with other remedies in such a manner as to secure more certainly our indication. Alterants may be variously combined with each other, to fulfil our indication. In acute affections, demanding their interposition, we unite tartarized antimony with calomel, or ipecacuanha and blue pill, that we may more effectively change or subvert morbid processes going on in the system.

Anodynes to abate morbid irritability, or suspend existing irritation, we combine with our alterants, that their action may not be frustrated by the excessive nervous mobility. Tonics may advantageously be combined with alterants in cachectic forms of disease. The sulphate of quinine, or infusions of qaassia, are well adapted to this design.

Another rule to be noticed is, that of selecting the particular preparation for the case in hand. Calomel and blue pill answer best in disturbed states of functions of the chylopoietic viscera, and in the majority of acute and chronic diseases, demanding the use of mercurials. But in affections of the skin, the corrosive muriate of mercury is incomparably the better article. Eight grains of this preparation, with eight grains of muriate of ammonia, is to be dissolved carefully in a small quantity of water, and made into sixty-four pills, by the addition of the crumb of bread. One pill is to be given three times a day, the patient taking at the same time a decoction a dulcamara, or sarsaparilla, to the extent of a pint a day.

We have seen so much good arise from a protracted employment of minute doses of corrosive sublimate, that we cannot too strenuously insist on the superior efficacy of this preparation of mercury. We believe it better, even in acute venereal affections, where there is a predisposition to scrophula, or any marked deterioration of health to give corrosive sublimate, in preference to either calomel or the blue pill. The corrosive sublimate is more stimulating than calomel, and when cautiously given, not so apt to affect the mouth. In the typhoid stage of fever, we regard the hydrargyrum cum creta, with or without Dover's powder, as the most eligible form of mercury. It acts as a mild alterant, and is well adapted to soothe the follicular ulceration which exists in that stage of fever. The doses should be small, and frequently repeated. Three, four, or six grains, with the same quantity of Dover's powder, exert a very favorable influence upon the irritated surface of the intestines, and act with ample power upon the biliary secretion.

In scrophulous affections, where there exists a deteriorated state of the peptic powers, as is most frequently the fact, we prefer giving a combination of iodine and iron. The iodine of iron, or Durand's solution of the protiodide of iron, is the best form of the medicine, in cases of scrophula, accompanied with deficient action. Ten drops of the solution is the medium dose. In visceral obstruction, or dropsical effusion, where we wish to administer a tonic alterant diuretic, the hydriodate of potash is the best preparation. The dose is from two to five grains, three times a day, or from twenty to sixty drops of a solution, made by dissolving thirty grains in an ounce of distilled water. These articles should be given in syrup; for though we have no reliance in the position assumed by Prof. Dungison, that sugar is an alterant, yet we believe that it acts favorably in two ways, when freely used in the form of good syrup in deteriorated constitutions—first, as a mild nutrient, and secondly, by protecting the mucous surface of the stomach from the irritation that might arise from the direct contact of the iodine.

Another rule which should be observed in the administration of alterants is a careful observance of regimen. Regimenal treatment must not be overlooked, whilst we are attempting to bring our patient under the alterative agency of medicinal substances. The quality as well as quantity of food must be made a matter of strict injunction on the part of the attending physician. But here the patient is most likely to array appetite and habit against the judgment of his medical adviser. The point is one of deep import, and pregnant with the issue of the trial instituted by the physician for the restoration of his patient's health, and should not be surrendered to the discretion of the sick man. Articles of diet may not offend the stomach of the patient, and yet be injurious to him, by affording too much stimulus to the chylopoietic organs, and by imparting a too abundant nutrition.

On the other hand, there are cases which call for a dietetic management of a decidedly nutritive and exciting nature. In cachectic, or depraved conditions of the body, such as that which is often witnessed in scrophulous patients, a course of living calculated to invigorate and nourish is demanded.

An additional rule in the practical application of alterants refers to the period of their administration. The criterion by which we are to be governed in acute disorders is the abatement of morbid action. This abatement is revealed in various ways, and through different signs of recovery.

Restoration of the secretions of the great emunctorial organs is a very significant manifestation, in acute affections, that our alterants have done their work in suspending the anormal state of the capillary vessels of the secreting tissues, which had been, either directly or indirectly, induced by the morbific cause. Upon a restoration of the secretions, the tongue becomes moist, and begins to clean off, the thirst declines, the heat and dryness of the surface recede, the renal discharge is augmented, and becomes more transparent, the bowels are acted on with more facility, and the alvine evacuations altered in color and consistency. Other symptoms are seen;—the sleep, which had been either totally, or to a great extent, interrupted, now returns, and the sense of weakness and misery which was so oppressive, is exchanged for the pleasing sensations of returning health. The pulse, which, during the acme of the disease, was increased in volume, in force and frequency, and disturbed in rhythm, falls back gradually to a more natural type of fulness, strength and regularity. The deranged sensations which accompanied the disease are disappearing, and the patient is now verging on the confines of convalescence.

Where these phenomena, or a part of them, arise upon the exhibition of alterants in acute attacks of disease, we congratulate ourselves that they have wrought their desired work, and in a majority of cases we decide correctly. In cases of doubtful termination, where we are not cheered by decided omens of returning health, we think it safest to push our mercurial alterant till there is established another indication. That indication, however, is too much relied upon by some practitioners. The demonstration about the mouth, that our alterative plan has told on the system, and, through the system, on the suffering organs, is very often an auspicious token. But mercury may create, by a misplaced action, a dry ulcerative soreness, on the buccal mucous tissue, which is any thing but a satisfactory proof of its curative power. This dry ulcerative soreness of the mouth is to be deprecated in all cases; and where it occurs, the danger of the case is much enhanced. In all such instances, the further use of the remedy should be desisted from, and either further depletion instituted, or tonics directed, as the symptoms may indicate the propriety of the evacuant, or invigorating method of treatment. In chronic cases, the successful issue of the application of alterants to disease, is marked by the recession of deranged sensations, disordered functions, and anormal vascular action. The lesions of secretion and of nutrition, which occur so frequently in chronic affections, are often the pathological phenomena which spring from protracted irritation. This irritation may be in the brain, lungs, or chylopoietic apparatus, or it may be constitutional. Latent irritation may persist with little functional disturbance of the organ affected by it; and another, either contiguous or remote part, may receive the full measure of the disturbance wrought. This element of chronic disease, makes the diagnosis one of extreme perplexity, in those instances in which we find a complication of morbid phenomena. The predisposition is to be carefully studied—whether hereditary, acquired, ætal, or sexual; the cause acting in the production of the malady noted, and also the point of sensible attack—in our endeavors to arrive at a sound judgment upon the related affections of the several parts involved in the morbid action.

The first appearance of the beneficial operation of alterants in chronic affections is that which is exhibited by the innervation. The deranged sensations which existed in the head, chest, abdomen, back and limbs are mitigated—the constitutional irritability is lessened—and the almost neuralgic state of the skin gradually leaves the patient. Pain in the head, either cephalalgia, or a species of tic douloreux, persistent, or intermittent, is a frequent symptomatic affection, dependent upon the state of the chylopoietic viscera. The rectification of the disordered function of the organs of nutrition will not always remove the pain in the head. There may supervene upon this protracted irritation of the brain, or its membranes, an inflammatory congestion, which requires either general, or local depletion, for its cure, whilst alterants are exhibited to correct the vitiated state of the digestive apparatus.

The point or surface to which we address our prescriptions hould not be disregarded. Where there is gastric disturbance, and our object is to influence the constitution by our alterants, we should make the skin the avenue for their communication to the suffering organ. Thus in liver affections we will find it best to employ inunction, or the nitro-muriatic bath, instead of giving calomel or blue pill internally.

The endermic medication, we believe, is too much neglected by physicians. The iatraleptic method, or that of application to the undenuded surface of the body, is one of great value in many instances. Iodine is frequently used in the form of ointment to discuss scrophulous and other indolent tumours.

The lungs are seldom made the avenue for the impartation of medicinal impressions; but we know that mercurial vapors being inhaled, will speedily bring the system under a mercurialism.

The final and most important rule to guide us in the exhibition of alterants, is that of adapting the medicinal article, in its curative relations, to the existing constitutional and local derangements. An article may bear general curative relations to a disease, and yet when prescribed without a due attention to the modifying circumstances, preceding and accompanying the existing state of the disease, it may act most injuriously. Mercury possesses strong curative relations for the venereal disease; so does iodine for scrophula, and so does arsenic for cutaneous affections; but were we to depend upon general therapeutics alone, without a careful analysis of the disease, and a rigorous application of special therapeutics to each case we should blunder into the most mischievous absurdities of practice, when we attempted the cure of every case of venereal with mercury, or of scrophula with iodine, or of diseases of the skin with arsenic.

Predisposition is to be studied, with the absolute or relative plethora existing; then the cause of the disease; then the seat occupied by it; after which we endeavor to discover what relations the disordered state of one organ bears to the deranged conditions of other parts, or of the whole system, and whether the original malady has been propagated from one point to other points by extension, remote changes, or substitution.

Besides, let the diligent and investigating physician reflect upon the satisfaction which he derives from the review of a case of difficulty which, by persevering pains-taking, has been enabled to solve, and satisfactorily treat. In order to make plain the views which we entertain on the difficulties which the clinical physician meets with in the elucidation of dubious pathological conditions of the system, and of particular organs, we will give some practical exemplifications of disease. We sail by a plain chart, under a comparatively cloudless sky, when we are called to treat open forms of fever, and easily recognized inflammations. But when the case is one of complication, and the symptoms obscurely announce the seat of disease, whilst we are assured that our patient is hastening with sure steps to the grave, then we feel the importance of having comprehensive and enlightened conceptions of general pathology. We will adduce cases of both acute and chronic affections to illustrate our positions.

A young woman, aged 18, of scrophulous diathesis, and of nervous temperament, complained of pain in the head, and of being very restless. She continued in the state of *malaise* for more than a week, when she took to bed; fever was present to a slight degree, and the pain continued in the head. She was bled and purged, with benefit. Her menstrual discharge came on, and with accustomed regularity flowed for several days. During the catamenial effort, she had a decided fit of hysterics, after which some coma supervened. She remained more or less comatose, two or three days; the pulse being undisturbed, the heat of the skin little above its natural state, the respiration tranquil, and bowels susceptible of being readily evacuated by purgative medicines. We saw her with three other medical gentlemen, on the seventh day of her confinement to bed. She had been blistered on the ankles, and on the back of the neck, and had been purged frequently with calomel and other cathartics. She lay with her eyes open, and turned up—striking the head board of the bed with one or both hands, or throwing them out in a child-like manner, as if to amuse herself. The pulse was sixty-five, and regular, when we first saw her; the tongue but little coated; the intelligence almost null; the head thrown back; the skin not hot; the *abdomen not tense or tender*. In consultation, it was agreed to give her nervines that day; at night the plan was altered, as a majority of the physicians in attendance thought the brain affected. The case grew worse; the pulse in a few days became rapid, and at times intermittent; and two or three days before she died the coma left her, and consciousness returned. Still the head was thrown back; and a day before death she had slight spasms of the muscles of the face, and quick tetanic contractions of the voluntary muscles generally.

*Post mortem.*—More than six ounces of water in the brain, both around the upper and lower portions of the hemispheres, and in the ventricles; tubercles diffused throughout the peritoneum; intense inflammation of the peritoneal sac in the pelvis, and lower part of the abdomen, with some inflammation in the mucous tissue of the rectum. Miliary tubercles studded the texture of the upper portion of both lungs, and drops of pus issued from the minute bronchi upon a section of the lungs, and there was a firm adhesion between the liver and diaphragm. This was a puzzling case, and one that was not comprehended aright, else the treatment would have been more energetic. There was a scrophulous as well as sexual predisposition in the case, which complicated its phenomena. Whether the disease originated in the brain, and subsequently affected the abdomen, or originated in the abdomen, and sympathetically irritated the brain, is yet *sub judice* among the medical attendants. That the inflammation commenced in the brain, and through the peculiar predisposition extended to the lungs and peritoneum, is the most plausible inference to our mind. After copious detractions of blood, generally and locally, such a case should have been treated by large doses of tartar emetic, with calomel. We have omitted the details of the treatment, such as small doses of calomel, with antimonial powder, cold applications to the shaved scalp, &c. Our remedial plan did not reach the morbid state of the capillary vessels of the brain and peritoneum. The tubercles were of recent production, as her health had been good to the period of her fatal attack.

There is no question but that dropsy of the brain supervenes often very rapidly upon abdominal irritation in scrophulous persons.

A young gentleman, aged 17, was a hard student, and grew rapidly—being tall and slender; whilst at college complained of pain in the abdomen, with general indisposition. He came home, and was placed under the care of his brother, an intelligent physician. There was no cough, nor did the stethoscope detect any lesion of the pulmonary apparatus; his tongue was red; and there was much irritation of the bowels, evinced by general abdominal tenderness, and pain upon pressure. The alvine evacuations were frequent and painful. These symptoms persisted for a few weeks, when cough came on, then night sweats, purulent sputa, and in a few months from his attack he died of phthisis pulmonalis. Here there was ætal predisposition, unaccompanied by any positive evidences of a strumous diathesis. The appropriate plan of treatment in such cases should be counter-irritation, with such alterants as ipecacuanha and blue pill, and mild gentle exercise in the open air. Study, and every other effort that would tax the mental and corporeal powers, should be most scrupulously avoided. An unirritating nutritious diet, and flannel to the skin, should not be omitted in conducting the cure. The ipecacuanha exercises a most salutary influence in procuring an abatement of irritation of the mucous tissues. We confide much in it in such cases as the above, and likewise in chronic bronchitis and asthma. It should never be given so as to sicken, or produce alvine evacuations.

Alterants never act favorably when they bring on nausea, or excite into prompt and energetic movement the functions of other organs. It is, therefore, better to give them after the patient has taken some food.

The above was a case of extension of disease from the abdomen to the lungs, grafted upon a predisposition under which the patient labored.

The following is an exemplification of remote changes grafted upon a scrophulous diathesis. A gentleman was very subject to diarrhœa, until the absorbent glands in the neck enlarged and suppurated; his bowel affection after that did not return. He cured up the glandular affection by taking Swaim's panacea, and by the use of topical remedies. The diarrhœa did not return; but in a few months the lungs became affected, tuberculous deposition took place in them, and he died of phthisis pulmonalis. The treatment in such a case should be similar to the last-mentioned.

The following are cases of substitution, or conversion, of disease. A child had measles, with great inflammation of the bronchial tubes, which yielded to an anti-phlogistic treatment. Soon after the disappearance of the eruption, dysentery in a very severe form came on, which lasted for several weeks. Upon the dysenteric symptoms being checked, dropsy of the abdomen supervened, with as entire a cessation of the dysenteric complaint as upon the supervention of dysentery the bronchitis had declined. Digitalis and calomel were given, which produced frequent watery discharges from the bowels, that induced so great a prostration as to jeopard the life of the patient; stimulants were freely given; after which the child slowly recovered. A man, lean and muscular, had an attack of acute inflammatory rheumatism, which was very obstinate in giving way under a depletory course of treatment. Upon visiting him one day, we found that his rheumatism had in a few days abated, and we congratulated the patient on the prospect of his speedy restoration. But we were sent for the next morning to prescribe for a new disease. Severe dysentery had come on, and the joints no longer were painful on motion. We combated the dysentery with calomel and Dover's powder, with counter-irritation over the abdomen; and in a few days it abated. Now, the rheumatism returned nearly as bad as previously, and the morbid action alternately passed from the bowels to the joints, four or five times, till the man, wearied with our medication, called in an emperic. In such cases, perhaps, our best plan would be to institute a decided mercurial impression, and maintain it for a few weeks. The thorough alterant action of mercury will arrest this tendency to metastasis, when no other remedy will. A conjunction of digitalis, or opium, to abate irritability of the system, is a happy one, and fulfils an important indication in such difficult pathological complications.

